# Evolution of oral and oropharyngeal cancer in the Basque Country (Spain) from 1986-1994 to 2012-2017

**DOI:** 10.4317/medoral.27518

**Published:** 2025-10-14

**Authors:** Iñaut Amezaga-Fernandez, Xabier Marichalar-Mendia, Irene Lafuente-Ibáñez-de-Mendoza, Arantza López-de-Munain-Marqués, José Manuel Aguirre-Urizar

**Affiliations:** 1 Oral Medicine and Pathology, Department of Stomatology, Faculty of Medicine and Nursery, University of the Basque Country (EHU/UPV), Spain; 2 Department of Nursery I, Faculty of Medicine and Nursery, University of the Basque Country (EHU/UPV), Spain; 3 Cancer Population Registry of the Basque Country, Department of Health, Basque Government

## Abstract

**Background:**

The Basque Country (Spain) is one of the European regions with the highest incidence of oral and oropharyngeal cancer, which still shows an unacceptably poor prognosis. The aim of this study was to update the epidemiological and prognostic data on oral cancer in the Basque Country and to analyse its evolution with respect to a previous study carried out in 1986-1994.

**Material and Methods:**

This retrospective study included all cases diagnosed with oral and oropharyngeal cancer in the Basque Country from 2012 to 2017, obtained from the Cancer Population Registry. Descriptive analysis of the epidemiological data on oral-oropharyngeal cancer and survival analysis of oral squamous cell carcinoma were performed.

**Results:**

A total of 1,762 cases of oral-oropharyngeal cancer were analysed. The crude incidence was 7.21 cases in women and 18.07 in men. We recognised an increase in females, older patients and gingival neoplasms. Squamous cell carcinomas accounted for 92.6% of the tumours, with 70.4% affecting males, 91.8% living in urban areas, and 50.2% diagnosed at advanced stages. The overall 5-year survival rate for oral-oropharyngeal cancer in the Basque Country was 50.2%.

**Conclusions:**

Oral and oropharyngeal cancer incidence in the Basque Country has decreased overall, although it shows a progressive increase in women, older people and tumours located in the gingiva. The prognosis of oral cancer in the Basque Country is still very poor, thus it is necessary to implement specific preventive and diagnostic protocols to improve it.

** Key words:**Oral cancer, oral squamous cell carcinoma, epidemiology, prognosis, survival, gingiva.

## Introduction

According to the Global Cancer Observatory, 389,846 cases of oral cancer were diagnosed worldwide in 2022, ranking as the 16th most frequent cancer [1]. The Basque Country (BC) has a high incidence of oral cancer, being the second autonomous community in Spain with the highest incidence of oral and oropharyngeal cancer, and ranking 13th out of 92 cancer registries in Europe [2]. We have known for some time that oral and oropharyngeal cancer is one of the most frequent malignant neoplasms in the BC [3], with an incidence per 100,000 inhabitants of 11.1 cases in women and 27.4 in men between the years 2015-2019 [4].

Oral squamous cell carcinoma (OSCC) is the most common oral cancer neoplasm, accounting for more than 90% of cases [5,6]. The remaining 10% is composed of haematolymphoid neoplasms, salivary gland neoplasms, sarcomas and metastases. In our environment, OSCC mainly affects men between 50-70 years of age, although in recent decades an increase in incidence has been observed in women [4]. Unfortunately, and despite the advances, OSCC still has a very poor prognosis, which has hardly improved in recent years [7-9]. The aggressiveness of this oral neoplasm, together with the high percentage of late diagnoses, and the high number of recurrences and second primaries, result in a 5-year survival rate of less than 50% [7,9].

Although several markers with prognostic value have been identified, the most important factor still accounts for the presence of cervicofacial lymph node metastases at the time of diagnosis, as well as the complete surgical elimination of the tumour with free margins [7,10,11]. Therefore, the only effective strategy currently available to improve the poor prognosis of OSCC remains its early diagnosis and treatment [7,10].

Despite the high incidence of oral cancer in the Basque Country, few studies have analysed the particular characteristics of this malignant pathology in the region, beyond general reports issued by the Basque Cancer Registry [4]. Only the study by Izarzugaza *et al*. [3] analysed in greater depth the data on oral and oropharyngeal cancer in the BC, based on cases diagnosed between 1986 and 1994, i.e. almost three decades ago.

With this background, we proposed to carry out a new study about oral cancer in the BC, in order to update its epidemiological, clinicopathological and prognostic characteristics, and to determine its importance in the region, to identify data which could help us prevent it and improve its prognosis.

## Material and Methods

We conducted a retrospective observational study, including all patients diagnosed with oral and oropharyngeal cancer in the Basque Country between 2012 and 2017, with a minimum follow-up time of 5 years.

- Patient data

Anonymised data on the cases included were obtained from the Cancer Population Registry of the Basque Country. Based on the International Classification of Diseases 10 (ICD-10), the following locations were included: lip (C00), base of tongue (C01), oral cavity (C02-C06), palatine tonsil (C09) and lateral and posterior walls of the oropharynx (C10.8-C10.9). A specific protocol was designed to collect epidemiological, clinicopathological, diagnostic and evolutionary data for each case.

The variables analysed were age, sex, residential area, histopathologic type of neoplasm, anatomical location, extension at the time of diagnosis (localised, regional, disseminated), death and cause of death. Dates of diagnosis, death and last follow-up were also recorded to calculate survival time. The residential setting was categorised as rural or urban based on its characterisation by the Basque Government in the three provinces of the Basque Autonomous Community (Araba, Bizkaia and Gipuzkoa). Histological type and cause of death were coded based on ICD-10. The study followed the Ethical Principles for Medical Research Involving Human Participants (Declaration of Helsinki).

- Statistical analysis

For the statistical analysis, the IBM SPSS Statistics software (version 28.0) was used. First, incidence of oral cancer in the BC was calculated, including crude rates and adjusted rates to the European standard population of 2013 and the world standard population of 2000-2025. Subsequently, a special descriptive analysis of OSCC was performed, excluding neoplasms of other origins. Frequencies and percentages were calculated for qualitative variables and mean and standard deviation (SD) for quantitative variables. A comparative analysis of OSCC was carried out between the three provinces of the BC (Araba, Bizkaia and Gipuzkoa) with the main variables, using the chi-square test. For the survival analysis of OSCC, Kaplan-Meier curves were constructed for overall survival (OS) and disease-specific survival (DSS). To identify factors related to OS and DSS, survival curves were compared using the log rank test. Statistical significance was established at *p-value*s ≤ 0.05.

## Results

- Oral and oropharyngeal cancer incidence in the Basque Country

During the study period (2012-2017), a total of 1,762 cases of oral cancer were diagnosed in the BC, 545 in women (30.9%) and 1,217 in men (69.1%), with a male:female ratio of 2.3:1. The mean age of the patients was 66.58 years (SD±12.90; range 21-103).

Incidence rates by sex and province are shown in Table 1. The crude incidence of oral cancer in the BC was 13.52 cases per 100,000 inhabitants, with the highest rate in the province of Bizkaia (14.75) and the lowest in Araba (11.71). Crude rate in women was 7.21 cases per 100,000, and 18.07 in men. The highest crude rate in both sexes was in Bizkaia, although the highest rate standardised to the European population in women was in Araba.

Considering anatomic location (Table 2), the highest incidence rate in men was on the tongue, followed by the oropharynx and lower lip. In women, the tongue and oropharynx were also the two locations with the highest incidence, but the gingiva was third.

The most frequent histopathological type was squamous cell carcinoma, with 1631 cases (92.6%) (Table 3). Malignant salivary gland neoplasms were in second place (3%), followed by haematolymphoid neoplasms (2.9%). In addition, 10 cases of sarcomas were diagnosed (0.6%), 3 of melanomas (0.2%), and 10 malignant neoplasms of unknown type (0.6%).

- General analysis of OSCC

This section describes the main characteristics of the 1,631 patients diagnosed with OSCC in the BC, excluding neoplasms of other origins. The variables analysed are shown in Table 3.

The mean age at the time of diagnosis of OSCC was 66.96 years (range 26-103; SD±12.34), with most patients being over 65 years of age (54.3%), and only 2.7% under 45 years of age. More than two thirds of the cases were diagnosed in men (70.4%). Bizkaia accounted for 57.6% of all cases of OSCC in the BC, and the majority (91.7%) resided in urban environments.

Regarding clinical aspects, the most frequent location was the lingual border (18.5%), followed by the lower lip (14.3%) and the palatine tonsil (12.8%). Regional lymph node involvement was present in 46.7% of cases at diagnosis, and distant metastases in 3.5%.

Histopathologically, the vast majority of cases were conventional squamous cell carcinomas (SCC) (95.6%), with verrucous carcinoma being the most common variant with 36 cases (2.2%), followed by basaloid carcinoma with 13 cases (0.8%). During the study period, 29.5% of patients died because of OSCC. Other neoplasms were the second cause of death (13.7%).

- Survival analysis of OSCC

**Figure 1:**
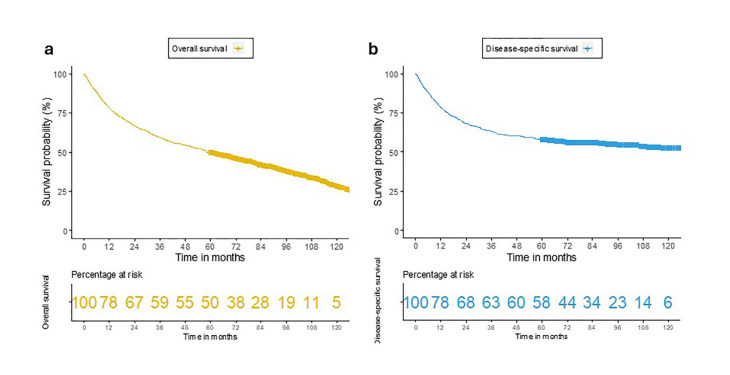
Oral-oropharyngeal SCC survival curves for a) overall survival; b) disease-specific survival.

In the survival analysis of OSCC in the BC, we obtained a 5-year OS of 50.2%, and a DSS of 57.8% (Fig. 1).

**Figure 2:**
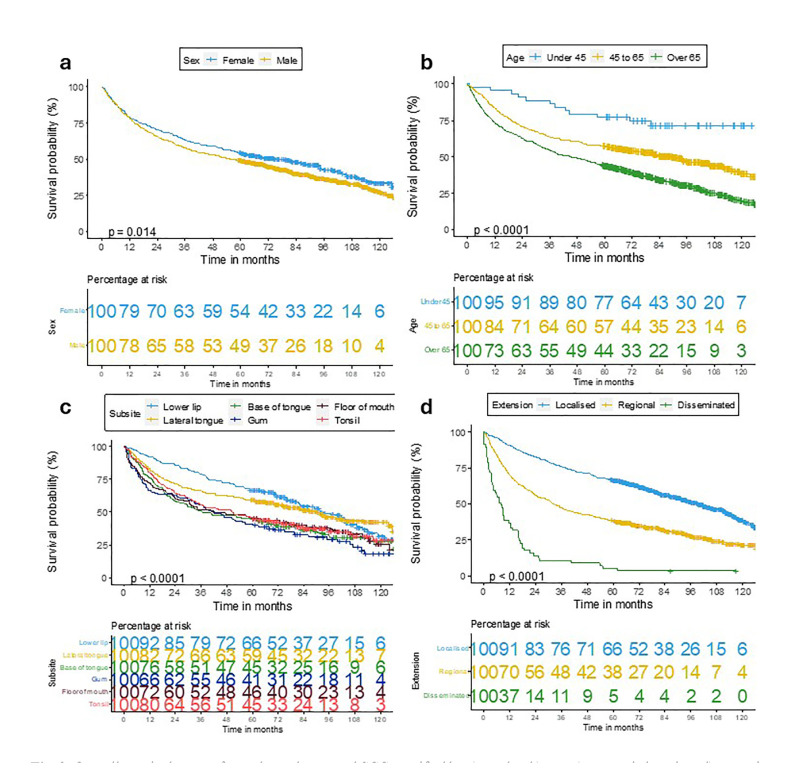
Overall survival curves for oral-oropharyngeal SCC stratified by a) gender; b) age; c) anatomic location; d) extension.

The variables that were significantly related with OS were sex, age, anatomic location and extension (Fig. 2). Women had a higher OS than men (*p*=0.014). Patients younger than 45 years had a better OS, while those older than 65 years had a worse survival. The subsite with the highest survival was the lower lip (66%), followed by the lingual border (59%), while the gingiva (41%) showed the lowest OS (*p*<0.001). Localised tumours had a better prognosis than those disseminated to regional lymph nodes and those with distant metastases (*p*<0.001).

**Figure 3:**
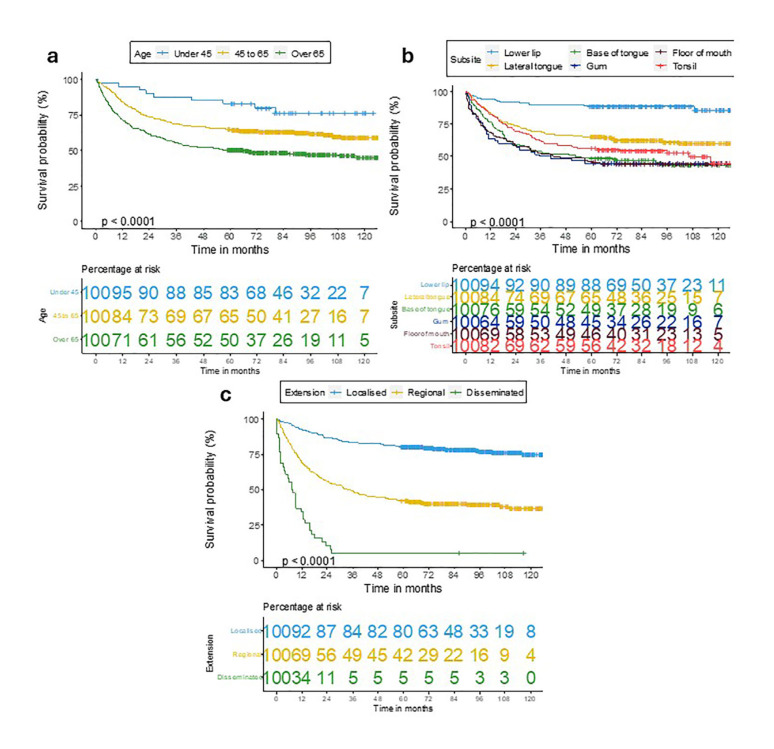
Disease-specific survival curves for oral-oropharyngeal SCC stratified by a) age; b) anatomic location; c) extension.

Regarding the analysis of factors related to DSS (Fig. 3), we obtained the same results for age (*p*<0.001), anatomical location (*p*<0.001) and extension (*p*<0.001).

## Discussion

Our study shows the importance of oral cancer in the Basque Country, which is one of the regions in Europe with the highest incidence of this malignant neoplasm [2]. In our previous study, carried out in Galicia (Spain) [7], we showed that OSCC had certain particularities in that region, especially a poor prognosis associated with late diagnosis and the advanced age of patients. The results of the present study show that oral cancer in the BC has a high incidence and some specific aspects, including an unaccepTable prognosis, which unfortunately shares with other regions of Europe and the world [9,11].

- Oral and oropharyngeal cancer incidence in the Basque Country

One of the main motivations for our study was to perform a comparative analysis with the previous work by Izarzugaza *et al*. [3], which researched oral cancer in the BC between 1986 and 1994. With that reference, oral and oropharyngeal cancer crude incidence in men has decreased from 24.1 to 18.07 cases per 100,000 inhabitants, whereas in women, this number has more than doubled, from 3.1 cases to 7.21 cases [3]. If we compare the male-female ratio, this tendency becomes even more evident, as it has changed from 7.4:1 (1986-1994) to 2.3:1 (2012-2017). We have also observed an increase in the mean age of patients, from 60.5 years in 1986-1994 [3] to 66.58 years in 2012-2017.

These findings would be related to the changes that have occurred with respect to the main oral carcinogenic factors in our environment. During the second half of the last century, there was an increase in tobacco consumption by women in Spain, rising from 0.9% in 1945 to 22.5% in 1995 [12]. Therefore, cases of OSCC diagnosed in the present study, mostly in those over 60 years of age, would correspond to this increase, which explains the rise of the incidence in women. However, over the last 30 years, tobacco consumption has decreased in both sexes and has tended to equalise, with current levels in the BC standing at 21.2% in men and 14.6% in women [13]. We believe that this decline will contribute to a decrease in the rate of tobacco-associated cancers in future generations, both oral-oropharyngeal and other.

With regard to alcohol and its relationship with oral carcinogenesis, consumption has remained unchanged in recent decades in the BC, with 73.2% of men and 62.7% of women reporting having consumed alcohol in the last month [14].

In relation to these tobacco-alcohol consumption data and the OSCC incidence evolution in the BC, it is to be expected that the number of cases among women will continue to increase in the upcoming decades, coming closer and closer to that of men. This makes it necessary to implement specific preventive measures aimed at reducing these carcinogenic habits, especially in the female gender.

In comparison to the incidence of cancer in the different oral locations by gender in the study by Izarzugaza *et al*. [3], a decrease was observed amongst men in almost all sites, with the greatest difference in lip cancer, which has gone from a rate of 5.8 in 1986-1994 [3], to a rate of 2.7 in 2012-2017. In women, the incidence has raised in all locations, especially in the gingiva, from 0.3 to 0.92, and in the floor of the mouth, from 0.2 to 0.6, which is three times the rates of 3 decades ago [3].

We believe that these changes are also related to social modifications of some etiopathogenic factors. Several studies [15-17] have pointed that tobacco consumption is specially related to floor of mouth SCC, and to a lesser extent with lingual margin SCC. In contrast, the gingiva is the oral cancer subsite least related to tobacco use, develops in older patients, and shows a correlation to specific oral premalignant disorders such as proliferative multifocal leukoplakia [15,18]. This observations may be associated with the increase in cases of gingival carcinomas in both sexes, which has already been observed by other authors [5,6]. To note, the gingiva currently represents the second most frequent location of OSCC in several regions worldwide [7,11,19]. All these data would be indicative of a changing trend in the classical epidemiology of OSCC, with an increasing development in women, at older ages and localised in special areas such as the gingiva [6,7,20].

As expected, the most frequent type of neoplasm in our study was squamous cell carcinoma, followed by salivary neoplasms and haematolymphoid cancers. This contrasts with the previous study by Izarzugaza *et al*. [3], in which the haematolymphoid neoplasms were the second most frequent malignant oral pathology.

- Specific analysis of oral SCC in the Basque Country

During the period 2012-2017, OSCC affected people with a mean age at diagnosis of 66.96 years, similar to that observed in other regions of Spain [7,19], but higher than in other regions of the world [11,18]. Also, the percentage of patients younger than 45 years was lower than in other studies [8,18]. These results could be related to the recognised general ageing of Basque population, with 23% of the inhabitants over 65 years of age, and to changes in oral carcinogenic factors (tobacco and alcohol) [13,14].

An important result of our study was the significant relationship between age and survival of OSCC in the BC, both overall and disease-specific. Young patients, under 45 years of age, had a better prognosis than those of intermediate age (45-65 years), and much better than those of advanced age (over 65 years). This association had already been recognised in previous studies [18,21], and could be related to the existence of poorer general health and comorbidities in older patients, leading to lower therapeutic effectiveness and higher mortality.

As previously mentioned, more than two-thirds of the patients with OSCC in our study were men, as has been widely recognised [6,8,10,11,15]. A relevant result has been that women had higher OS than men, similar to what has been observed in other studies [7,18,22]. These results could be related to a shorter diagnostic delay of OSCC in women, as has been reported [23].

The majority of OSCC patients in the BC reside in urban settings, which contrasts with our previous study carried out in Galicia [7]. We believe that this data depends mainly on the demographic and socioeconomic characteristics of each region.

The most frequent anatomic locations of OSCC in our study (lingual border, floor of mouth and gingiva) were the same as in other studies [7,8,19]. Interestingly, we observed a significant relationship between OSCC location and survival. Lip cancer had the best prognosis, followed by lingual border SCC, with the worst survival for gingival SCC. Some authors [24,25] are in line with these results, but others [26,27] report better survival for gingival SCC. These contradictory results could be related to the different carcinogens associated with each population studied and the advanced age of these patients.

Although we know that early diagnosis is currently the only effective strategy to improve prognosis in oral cancer, in our work recognised that more than half of the cases were still diagnosed at advanced stages, as unfortunately reported in other studies [8-10,15]. As expected, localised cancers had higher OS and DSS than disseminated cancers.

When analysing the cause of death, it is striking that the second most frequent cause of death were other cancers, which is consistent with previous studies [28]. Jiang *et al*. reported that 32.25% of patients with died from other malignant neoplasms, also being the second most frequent cause of death. This could be explained by the higher risk of these patients to develop other primary malignant tumours, especially of the upper aerodigestive tract, associated with similar carcinogenic factors [29].

Regarding prognosis, we can point out that survival of OSCC in the Basque Country remains poor, not exceeding 50% at 5 years, similar to that observed in other regions of Europe and the world [6-10]. We believe that this poor outcome is due to the high percentage of late diagnoses in this neoplastic pathology, together with the high biological aggressiveness of this carcinoma and the reduced effectiveness of current treatments. Furthermore, the advanced age of many of the patients could be related to this poor survival. For all these reasons, it is necessary to design and implement specific preventive and diagnostic programmes to avoid its development and improve its prognosis.

This study has a series of limitations that must be taken into account. The retrospective design entails the loss of some data, as well as the impossibility of obtaining variables not collected in the Basque Cancer Registry. In particular, it was not possible to access information on TNM staging in many of the cases. Nevertheless, the large number of patients included in the study, which covers the total number of cases of oral cancer diagnosed in the BC, we believe that it provides representative and up-to-date information on the status of this malignant neoplasm in our region, which is useful for the design of preventive and early diagnosis programmes.

## Conclusions

The epidemiology of oral cancer in the Basque Country has changed over the last 25 years, with a rising incidence in women, in older patients and in tumours located in the gingiva. The prognosis of OSCC in the Basque Country is poor, with a 5-year overall survival of 50.2%, which would be related to its late diagnosis, biological aggressiveness and advanced age of patients. Lip and lingual border SCC show the best prognosis, while gingival SCC has the lowest survival. Epidemiological changes and the persistence of poor prognosis make it necessary to design and implement specific preventive and diagnostic protocols for this malignant neoplasm in the BC.

## Figures and Tables

**Table 1:** Total oral cancer cases and incidence per 100,000 inhabitants in the BC during the 2012 to 2017 period. ASR: Age Standardised Rate; BC: Basque Autonomous Country.

Region	Category	n	Crude rate	ASR (Europe)	ASR (World)
BC	Female	545	7.21	6.94	3.86
	Male	1217	18.07	19.34	10.72
	Total	1762	13.52	12.6	7.07
Araba	Female	76	7.79	7.14	4.20
	Male	150	15.70	16.34	9.16
	Total	226	11.71	11.47	6.58
Bizkaia	Female	303	8.52	6.99	3.76
	Male	710	21.43	21.42	11.61
	Total	1013	14.75	13.38	7.37
Gipuzkoa	Female	166	7.65	6.57	3.81
	Male	357	17.13	17.20	9.94
	Total	523	12.29	11.54	6.73

**Table 2:** Total cases and crude incidence rates per 100,000 inhabitants for oral cancer in the BC by subsite and gender during the 2012 to 2017 period.

Location	Female		Male	
	n	Crude rate	n	Crude rate
Tongue	149	2.2	371	5.84
Oropharynx	94	1.40	279	4.4
Lip	61	0.9	174	2.74
Floor of mouth	40	0.6	125	1.97
Gingiva	62	0.92	45	0.71
Other	139	2.07	223	3.51

**Table 3:** Epidemiological and clinical data of oral SCC. SCC: squamous cell carcinoma.

Variable	Category	n (%)
Age	<45 years	44 (2.7)
	45 - 65 years	702 (43.0)
	>65 years	885 (54.3)
Gender	Female	483 (29.6)
	Male	1148 (70.4)
Residential setting	Urban	1484 (91.7)
	Rural	135 (8.3)
Anatomic location	Lingual border	302 (18.5)
	Floor of mouth	162 (9.9)
	Gingiva	98 (6.0)
	Lower lip	233 (14.3)
	Tonsil	209 (12.8)
	Base of tongue	173 (10.6)
	Other	454 (27.8)
Extension	Localised	755 (46.3)
	Regional	762 (46.7)
	Disseminated	57 (3.5)
	Unspecified	57 (3.5)
Histologic subtype	Conventional SCC	1560 (95.6)
	Verrucous carcinoma	36 (2.2)
	Basaloid SCC	13 (0.8)
	Spindle cell SCC	6 (0.4)
	Papillary SCC	4 (0.2)
	Adenosquamous SCC	3 (0.2)
	Lymphoepithelial SCC	2 (0.1)
	Other	7 (0.4)
Cause of death	Oral SCC	495 (29.5)
	Other cancers	229 (13.7)
	Other causes	181 (10.7)
	Unspecified	106 (6.4)
